# Evaluation of lytic bacteriophages for control of multidrug-resistant *Salmonella* Typhimurium

**DOI:** 10.1186/s12941-017-0237-6

**Published:** 2017-09-22

**Authors:** Lae-seung Jung, Tian Ding, Juhee Ahn

**Affiliations:** 1 Department of Medical Biomaterials Engineering, Kangwon National University, Chuncheon, Gangwon 24341 Republic of Korea; 2 Department of Food Science and Nutrition, Zhejiang Key Laboratory for Agro-Food Processing, Zhejiang University, Hangzhou, 310058 Zhejiang China; 3 Institute of Bioscience and Biotechnology, Kangwon National University, Chuncheon, Gangwon 24341 Republic of Korea

**Keywords:** *Salmonella* Typhimurium, Bacteriophage, Antibiotic resistance, Lytic activity, Ciprofloxacin

## Abstract

**Background:**

The emergence of antibiotic-resistant bacteria can cause serious clinical and public health problems. This study describes the possibility of using bacteriophages as an alternative agent to control multidrug-resistant *Salmonella* Typhimurium.

**Methods:**

The potential lytic bacteriophages (P22-B1, P22, PBST10, PBST13, PBST32, and PBST 35) were characterized by morphological property, heat and pH stability, optimum multiplicity of infection (MOI), and lytic activity against *S*. Typhimurium KCCM 40253, *S*. Typhimurium ATCC 19585, ciprofloxacin-induced antibiotic-resistant *S*. Typhimurium ATCC 19585, and *S*. Typhimurium CCARM 8009.

**Results:**

P22-B1 and P22 belong to Podoviridae family and PBST10, PBST13, PBST32, and PBST 35 show a typical structure with polyhedral head and long tail, belonging to Siphoviridae family. *Salmonella* bacteriophages were highly stable at the temperatures (< 60 °C) and pHs (5.0–11.0). The reduction rates of host cells were increased at the MOI-dependent manner, showing the highest reduction rate at MOI of 10. The host cells were most effectively reduced by P22, while P22-B1 showed the least lytic activity. The ciprofloxacin-induced antibiotic-resistant *S*. Typhimurium ATCC 19585, and clinically isolated antibiotic-resistant *S*. Typhimurium CCARM 8009 were resistant to ciprofloxacin, levofloxacin, norfloxacin, and tetracycline. P22 showed the highest lytic activity against *S*. Typhimurium KCCM 40253 (> 5 log reduction), followed by *S*. Typhimurium ATCC 19585 (4 log reduction) and ciprofloxacin-induced antibiotic-resistant *S*. Typhimurium ATCC 19585 (4 log reduction).

**Conclusion:**

The results would provide vital insights into the application of lytic bacteriophages as an alternative therapeutics for the control of multidrug-resistant pathogens.

## Background

Over the last few decades, the repeated misuse and overuse of antibiotics has accelerated the emergence of antibiotic-resistant bacteria, leading to serious clinical and public health problems [1, 2]. The rapid spread of antibiotic resistance has left the development of new antibiotics due to the long and expensive clinical testing [3]. The antibiotic resistance of *Salmonella* species is mainly acquired by the production of antibiotic-degrading enzymes, alteration in membrane permeability, and activation of multidrug efflux pumps [4]. Recently, the prevalence of multidrug-resistant (MDR) *Salmonella* serotypes has increased the failure in antibiotic treatments [5–7]. The MDR *Salmonella* infection has become a global public health concern due to the annual increase in morbidity and mortality rates [8]. The therapeutic limitation of current antibiotics has added to the difficulty in treating multidrug-resistant bacterial infections. Hence, the development of alternative therapeutic treatments over antibiotics is essential for controlling multidrug-resistant bacteria.

Bacteriophage has received much attention as a possible alternative due to the specificity and self-replicating property with no adverse effects on beneficial microflora and human cells [9]. The specificity to target bacteria is attributed to the binding ability of bacteriophages to host cell surface receptors such as flagella, capsule, slime layer, lipopolysaccharides, and outer membrane proteins, resulting in the lysis of bacteriophage-infected bacteria expressed as lytic activity [10, 11]. The bacteriophage-binding receptors on the host cell surface can be altered though the modification of outer membrane components [12–14]. However, there is relatively little knowledge on the interaction between bacteriophages and multidrug-resistant bacteria in terms of the alteration in host cell surface receptors. Therefore, the bacteriophages are not directly applicable to multidrug-resistant bacteria. For the successful application of bacteriophage, this study was aimed to evaluate the lytic activity of potential *Salmonella* bacteriophages (P22-B1, P22, PBST10, PBST13, PBST32, and PBST 35) against *Salmonella enterica* serovar Typhimurium KCCM 40253, *S*. Typhimurium ATCC 19585, ciprofloxacin-induced antibiotic-resistant *S*. Typhimurium ATCC 19585, and *S*. Typhimurium CCARM 8009, having different antibiotic resistance profiles.

## Methods

### Bacterial strains and culture conditions

Strains of *S.* Typhimurium KCCM 40253, *S*. Typhimurium ATCC 19585, and *S*. Typhimurium CCARM 8009 were purchased from Korean Culture Center of Microorganism (KCCM, Seoul, Korea), American Type Culture Collection (ATCC, Manassas, VA, USA), and Culture Collection of Antibiotic Resistant Microbes (CCARM, Seoul, Korea), respectively. *Escherichia coli* ATCC 25922 was used as control strain to evaluate the antibiotic susceptibility. All strains were cultured in trypticase soy broth (TSB) (BD, Becton, Dickinson and Co., Sparks, MD, USA) at 37 °C for 20 h. The cultured cells were harvested by centrifugation at 3000×*g* for 20 min at 4 °C, washed twice with phosphate-buffered saline (PBS, pH 7.2), and diluted to 10^8^ CFU/ml prior to use.

### Stepwise selection method

The strain of *S*. Typhimurium ATCC 19585 was exposed to ciprofloxacin to induce antibiotic-resistant isogenic strain according to a serial passage assay [15]. *Salmonella* Typhimurium ATCC 19585 strain was cultured repeatedly in TSB and TSA by increasing ciprofloxacin concentrations from 0.03 to 1 µg/ml. The ciprofloxacin-induced antibiotic-resistant *S*. Typhimurium ATCC 19585 were stable for more than 10 passages in antibiotic-free TSB. The strain was cultured in ciprofloxacin-free TSB at 37 °C for 20 h prior to use.

### Bacteriophage propagation

*Salmonella* bacteriophages, P22-B1 and P22, were purchased from ATCC and PBST10, PBST13, PBST32, and PBST 35 were obtained from Bacteriophage Bank at Hankuk University of Foreign Studies (Yongin, Gyeonggi, Korea). The bacteriophages were propagated at 37 °C for 24 h in TSB containing *S*. Typhimurium KCCM 40253. The propagated bacteriophages were centrifuged at 5000×*g* for 10 min, filtered through a 0.2-μm filter to eliminate bacterial lysates, and further purified using a polyethylene glycol (PEG) precipitation assay. The bacteriophage titers were determined by using a soft-agar overlay method [16]. In brief, the collected bacteriophages were serially (1:10) diluted with PBS and gently mixed with the host cells (10^7^ CFU/ml) in TSB (0.5% agar). The mixture was poured over the pre-warmed base agar lawn. The plates were incubated at 37 °C for 24 h to enumerate the bacteriophages expressed as plaque-forming unit (PFU).

### Morphological assay

The morphological properties of *Salmonella* bacteriophages were determined by transmission electron microscope (TEM, LEO 912AB Omega; Carl Zeiss NTS GmbH, Oberkochen, Germany), located at the Korea Basic Science Institute (KBSI; Gangwon, Korea). The bacteriophages were transferred to the surface of carbon-coated copper film and negatively stained with 5% aqueous uranyl acetate (pH 4.0). After air-drying, the stained bacteriophages were observed under TEM (120 kV; 125,000× magnification).

### Heat and pH stability of bacteriophages

The susceptibility of bacteriophages to heat was evaluated at the range of 30–80 °C for 30 min. For isothermal treatment, 0.1 ml of each *Salmonella* bacteriophage (5 × 10^5^ PFU/ml) was inoculated into a pre-heated tube containing 9.9 ml of TSB and then treated at each target temperature for 30 min. After heat treatment, each tube was immediately cooled in an ice-bath. The pH stability of bacteriophages was evaluated in phosphate buffered saline (PBS, pH 7.2) adjusted to pHs ranged from 2 to 12. Each *Salmonella* bacteriophage (5 × 10^5^ PFU/ml) was exposed to different pH levels at 37 °C for 30 min. The pH-treated bacteriophages were immediately diluted with PBS to avoid further inactivation. After heat or pH treatment, viable bacteriophages were enumerated by using a soft-agar overlay assay.

### Determination of optimum multiplicity of infection

The lytic ability of *Salmonella* bacteriophages against *S*. Typhimurium KCCM 40253 (10^5^ CFU/ml) was evaluated at different multiplicity of infections (MOIs) ranging from 0.01 to 10. The host cells infected by each *Salmonella* bacteriophage were incubated at 37 °C for 6 h. After incubation, the host cells were enumerated using an Autoplate^®^ Spiral Plating System (Spiral Biotech Inc., Norwood, MA, USA) and a QCount^®^ Colony Counter (Spiral Biotech Inc.). The reduction rate was estimated by comparing with the number of the control cells treated without bacteriophages.

### Antibiotic disc susceptibility assay

The susceptibility of host strains, *S*. Typhimurium KCCM 40253, *S*. Typhimurium ATCC 19585, ciprofloxacin-induced antibiotic-resistant *S*. Typhimurium ATCC 19585, and *S*. Typhimurium CCARM 8009, was evaluated by using an agar disc diffusion assay. Each host strain was diluted to the turbidity of 0.5 McFarland standard and spread on Mueller–Hinton agar plate. Antibiotic discs used in this study were ampicillin (10 µg), cefotaxime (30 µg), cephalothin (30 µg), chloramphenicol (30 µg), ciprofloxacin (5 µg), kanamycin (30 µg), levofloxacin (5 µg), meropenem (10 µg), nalidixic acid (30 µg), norfloxacin (10 µg), sulfamethoxazole/trimethoprim (1.25/23.75 µg), and tetracycline (30 µg). The antibiotic discs were placed on Mueller–Hinton agar plate and then incubated at 37 °C for 20 h. The clear zone was measured to evaluate the antibiotic susceptibility. The susceptibility (S) and resistant (R) strains were defined as compared with the susceptibility of the control strain, *E*. *coli* ATCC 25922.

### Bacteriophage spotting assay

The lytic ability of *Salmonella* bacteriophages against *S*. Typhimurium KCCM 40253, *S*. Typhimurium ATCC 19585, ciprofloxacin-induced antibiotic-resistant *S*. Typhimurium ATCC 19585, and *S*. Typhimurium CCARM 8009 was determined using a spotting test. The serially (1:10) diluted bacteriophages (2 µl each) were spotted onto the soft-agar plates and incubated at 37 °C for 24 h. The formation of clear zone was determined to express the lytic capability.

### Lytic activity of bacteriophage

*Salmonella* bacteriophages were used to evaluate the lytic activity against *S*. Typhimurium KCCM 40253, *S*. Typhimurium ATCC 19585, ciprofloxacin-induced antibiotic-resistant *S*. Typhimurium ATCC 19585, and *S*. Typhimurium CCARM 8009. The host strains (10^5^ CFU/ml each) were mixed with bacteriophage (10^5^ PFU/ml) at MOI of 1 and incubated at 37 °C for 12 h. After 12-h incubation, the cultures were centrifuged at 3000×*g* for 20 min. The collected cells were serially diluted (1:10) with PBS. The proper dilutions were plated on TSA using an Autoplate^®^ Spiral Plating System (Spiral Biotech Inc.). After 24-h incubation, the viable cells were enumerated using a QCount^®^ Colony Counter (Spiral Biotech Inc.). The lytic activity was expressed as log N_c_/N_p_; N_c_ and N_p_ denote the counts of bacterial host cells treated without and with bacteriophages, respectively, after 12-h incubation.

### Statistical analysis

All analyses were performed in duplicate on three replicates. Data were analyzed using the Statistical Analysis System software (SAS 9.4; SAS Institute Inc., Cary, NC, USA). The general linear model (GLM) and least significant difference (LSD) procedures were used to compare means at p < 0.05.

## Results

### Morphological and physiological properties of various *Salmonella* bacteriophages

**Fig. 1:**
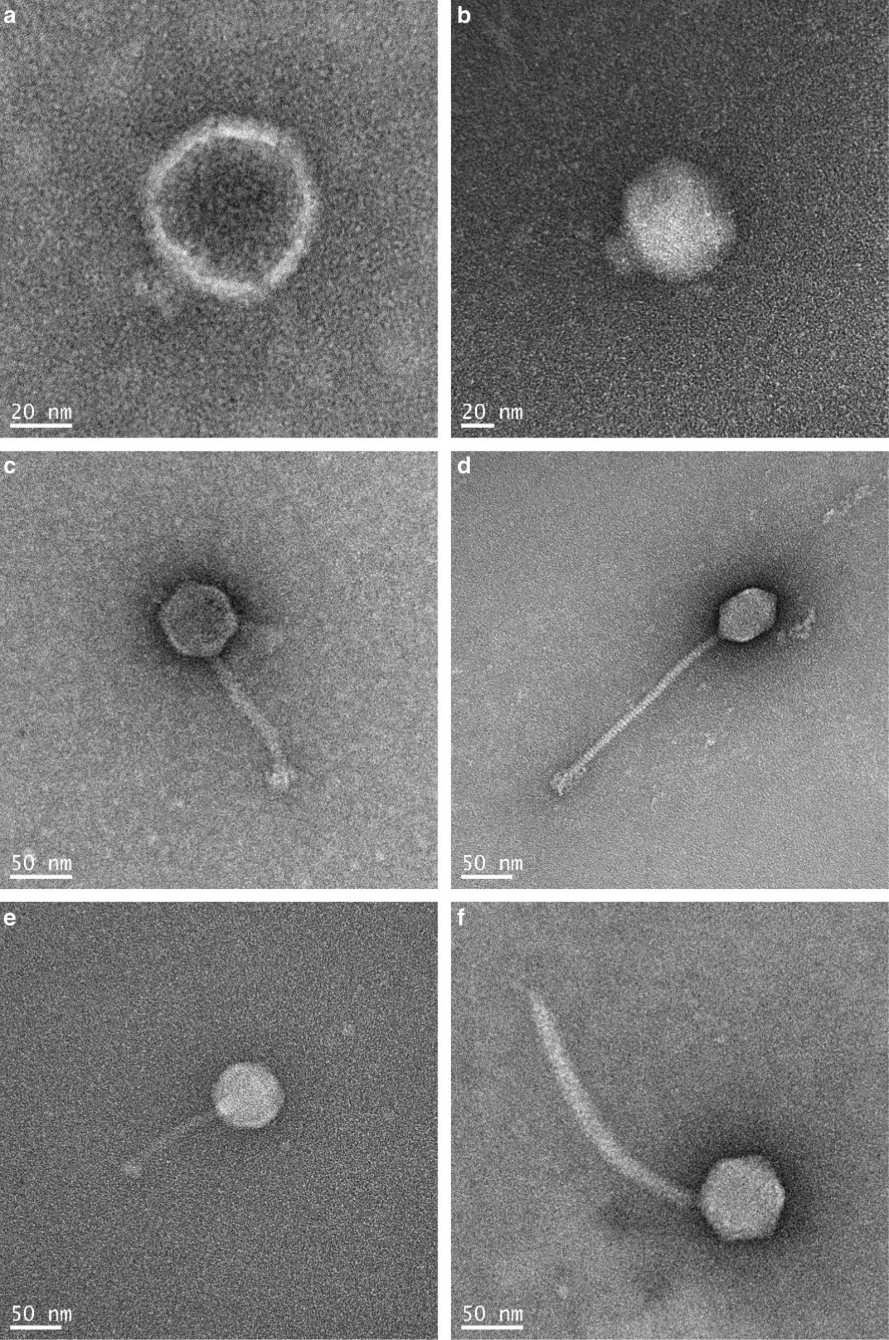
Morphology of bacteriophages; P22-B1 (**a**), P22 (**b**), PBST10 (**c**), PBST13 (**d**), PBST32 (**e**), and PBST35 (**f**)

The morphological characteristics of *Salmonella* bacteriophages (P22-B1, P22, PBST10, PBST13, PBST32, and PBST 35) were evaluated using TEM (Fig. 1). P22-B1 (Fig. 1a) and P22 (Fig. 1b) have regular icosahedral heads of 50 and 77 nm with short tails, respectively, belonging to the Podoviridae family. PBST10 (Fig. 1c), PBST13 (Fig. 1d), PBST32 (Fig. 1e), and PBST35 (Fig. 1f) show a regular polyhedral structure with heads of 45–66 nm and long tails of 94–186 nm, belonging to Siphoviridae family.

**Fig. 2:**
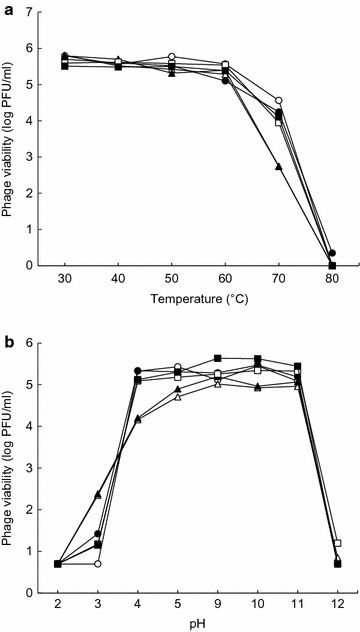
Susceptibility of *Salmonella* bacteriophages (○, P22-B1; ●, P22; □, PBST10; ■, PBST13; ∆, PBST32; ▲, PBST35) to heat (**a**) and pH (**b**)

The heat sensitivity of *Salmonella* bacteriophages was evaluated at the temperature ranged from 30 to 80 °C (Fig. 2a). All bacteriophages were stable to heat up to 60 °C, but the numbers of bacteriophages were reduced to below the detection limit at 80 °C. The sensitivity of *Salmonella* bacteriophages to pH was evaluated at different pH values ranged from 2 to 12 (Fig. 2b). P22-B1, P22, PBST32, and PBST 35 were stable at pHs ranged from 4 to 11, showing no noticeable reduction in the viable bacteriophage counts.

**Fig. 3:**
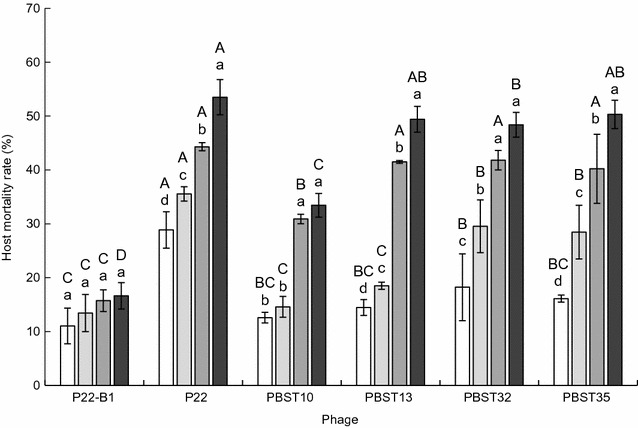
Reduction rate (%) of *Salmonella* Typhimurium KCCM 40253 treated with bacteriophages at different multiplicity of infections (MOIs) of 0.01 (□), 0.1 (), 1 (), and 10 (■). Bars with different uppercase (A–D) among bacteriophages and lowercase (a–d) within MOIs are significantly different at *p* < 0.05

The ability of *Salmonella* bacteriophages to lyse the host cells (*S*. Typhimurium KCCM 40253) was evaluated at different MOIs of 0.01, 01, 1, and 10 (Fig. 3). The reduction rates of *S*. Typhimurium KCCM 40253 were increased with increasing the MOI. The highest reduction rate of host cells was 54% for P22 at MOI of 10, followed by PBST35, PBST13, and PBST32. P22 was most effective on the reduction of host cells, whereas the least lytic activity was observed for P22-B1 at all MOIs.

### Antibiotic susceptibility and bacteriophage host ranges of *S.* Typhimurium

The antibiotic susceptibility of *S*. Typhimurium KCCM 40253, *S*. Typhimurium ATCC 19585, ciprofloxacin-induced antibiotic-resistant *S*. Typhimurium ATCC 19585, and clinically isolated antibiotic-resistant *S*. Typhimurium CCARM 8009 was determined using a disc diffusion assay (Table 1). *Salmonella* Typhimurium KCCM 40253 and *S*. Typhimurium ATCC 19585 were sensitive to most antibiotics. The susceptibility of *S*. Typhimurium ATCC 19585 to cephalothin, ciprofloxacin, levofloxacin, nalidixic acid, norfloxacin, and tetracycline was decreased after ciprofloxacin induction. The clinically isolated antibiotic-resistant *S*. Typhimurium CCARM 8009 was resistant to ampicillin, ciprofloxacin, kanamycin, levofloxacin, norfloxacin, sulfamethoxazole/trimethoprim, and tetracycline.

**Table 1:** Antibiotic disc diffusion (mm) of *Salmonella* Typhimurium strains

Antibiotic disc	*S*. Typhimurium KCCM40253	*S*. Typhimurium ATCC19585	*S*. Typhimurium ATCC19585-CIP	*S*. Typhimurium CCARM8009
AMP	27.4 ± 0.5 (S)	24.3 ± 0.5 (S)	22.4 ± 0.7 (S)	6.00 (R)
CEF	33.3 ± 0.9 (S)	31.5 ± 0.4 (S)	28.8 ± 0.6 (S)	32.0 ± 0.5 (S)
CEP	24.8 ± 0.8 (S)	24.8 ± 0.8 (S)	22.7 ± 0.4 (R)	25.2 ± 0.5 (S)
CHL	27.1 ± 0.4 (S)	25.7 ± 0.3 (S)	24.9 ± 0.7 (S)	23.5 ± 0.3 (S)
CIP	34.6 ± 0.6 (S)	33.2 ± 0.7 (S)	30.7 ± 1.0 (R)	30.9 ± 0.7 (R)
KAN	21.8 ± 0.9 (S)	21.8 ± 0.3 (S)	21.4 ± 0.6 (S)	6.00 (R)
LEV	31.0 ± 0.5 (S)	31.6 ± 0.5 (S)	27.4 ± 0.9 (R)	28.3 ± 0.5 (R)
MER	34.2 ± 0.5 (S)	32.3 ± 0.2 (S)	31.8 ± 1.2 (S)	32.5 ± 0.7 (S)
NAL	24.6 ± 0.9 (R)	23.3 ± 0.6 (R)	6.00 (R)	23.6 ± 0.7 (R)
NOR	32.5 ± 0.7 (S)	31.8 ± 0.5 (S)	28.6 ± 0.6 (R)	29.5 ± 0.4 (R)
SMA/TMP	23.6 ± 0.6 (S)	25.7 ± 1.0 (S)	24.4 ± 0.5 (S)	22.7 ± 0.5 (R)
TET	18.6 ± 0.8 (R)	19.3 ± 0.6 (S)	17.4 ± 0.2 (R)	7.5 ± 0.1 (R)

S and R indicate susceptible and resistant strain. AMP, ampicillin (10 µg); CEF, cefotaxime (30 µg); CEP, cephalothin (30 µg); CHL, chloramphenicol (30 µg); CIP, ciprofloxacin (5 µg); KAN, kanamycin (30 µg); LEV, levofloxacin (5 µg); MER, meropenem (10 µg); NAL, nalidixic acid (30 µg); NOR, norfloxacin (10 µg); SMA/TMP, sulfamethoxazole/trimethoprim (1.25/23.75 µg); TET, tetracycline (30 µg)

**Fig. 4:**
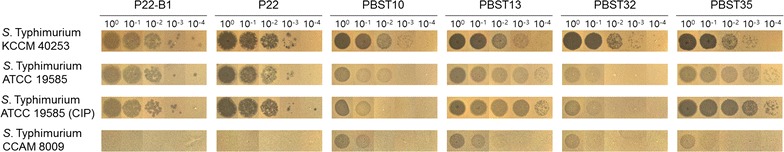
Lytic ability of bacteriophages (P22-B1, P22, PBST10, PBST13, PBST32, and PBST35) against *Salmonella* Typhimurium KCCM 40253, *S*. Typhimurium ATCC 19585, ciprofloxacin-induced antibiotic-resistant *S*. Typhimurium ATCC 19585, and clinically isolated antibiotic-resistant *S*. Typhimurium CCARM 8009

The host range of bacteriophages (P22-B1, P22, PBST10, PBST13, PBST32, and PBST 35) was determined using a spotting assay (Fig. 4). All bacteriophages tested in this study showed the specificity to *S*. Typhimurium KCCM 40253, *S*. Typhimurium ATCC 19585, and ciprofloxacin-induced antibiotic-resistant *S*. Typhimurium ATCC 19585. No host specificity was observed for P22-B1 and P22 against clinically isolated antibiotic-resistant *S*. Typhimurium CCARM 8009, showing no clear plaques on the soft agar.

### Lytic activity of bacteriophages against antibiotic-resistant *Salmonella* Typhimurium

**Fig. 5:**
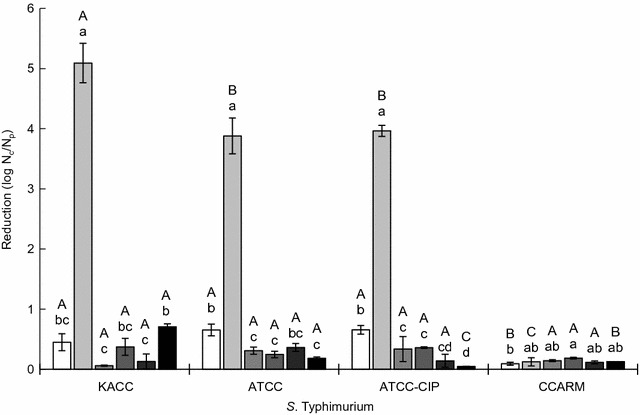
Lytic activity of *Salmonella* bacteriophages (□, P22-B1; , P22; , PBST10; , PBST13; , PBST32; ■, PBST35) against *Salmonella* Typhimurium KCCM 40253 (KACC), *S*. Typhimurium ATCC 19585 (ATCC), ciprofloxacin-induced antibiotic-resistant *S*. Typhimurium ATCC 19585 (ATCC-CIP), and clinically isolated antibiotic-resistant *S*. Typhimurium CCARM 8009 (CCARM). N_c_ and N_p_ indicate the numbers of host cells treated without and with bacteriophages, respectively, after 12-h incubation. Bars with different uppercase (A–C) among host strains and lowercase (a–d) within bacteriophages are significantly different at *p* < 0.05

The lytic activity of bacteriophages against various host strains was evaluated as shown in Fig. 5. The highest lytic activity of P22 was observed against *S*. Typhimurium KCCM 40253 (> 5 log reduction), followed by *S*. Typhimurium ATCC 19585 and ciprofloxacin-induced antibiotic-resistant *S*. Typhimurium ATCC 19585. The clinically isolated antibiotic-resistant *S*. Typhimurium CCARM 8009 showed the highest resistance to *Salmonella* bacteriophages (P22-B1, P22, PBST10, PBST13, PBST32, and PBST 35).

## Discussion

This study demonstrates the lytic activity of selected bacteriophages against multidrug-resistant *S*. Typhimurium strains, including antibiotic-induced resistant and clinically isolated strains. In order to compare the host specificity depending on the interaction between bacteriophages and host receptors, the isogenic strain was used to induce the antibiotic resistance. The application of bacteriophages can be one possible approach as an alternative agent to control multidrug-resistant *S.* Typhimurium.

The heat stability of PBST10 and PBST13 was significantly decreased at 70 °C when compared to other bacteriophages (Fig. 2a). PBST10 and PBST13 were relatively more stable than other bacteriophages at pH 3 but less stable at pH above 4 (Fig. 2b). All bacteriophages were highly sensitive to pH 2 and pH 12 (Fig. 2b). This is in good agreement with previous study showing most bacteriophages are highly stable at pHs between 4 and 9 [17, 18]. The observations suggest that the adsorption rates of *Salmonella* bacteriophages tested in this study may not be changed at the broad ranges of temperature and pH, which are the important environmental factors for the lytic activity of bacteriophages [19].

The different lytic activities of P22 and P22-B1 were observed depending on MOI (Fig. 3). The interaction of bacteriophages and host cells plays an important role in practical application. The efficacy of lytic activity is associated with the optimum ratio of bacteriophage to host cells, which leads to the increase in the binding chance of bacteriophages to the host cells [20]. The primary step in bacteriophage infection process is the attachment and adsorption, which contribute to the host specificity [10, 11]. Unlike the host cell reduction, the highest phage titers were observed at low MOI (data not shown). The result is due to the lysis-from-without phenomenon that the lysis of host cells can occur without bacteriophage multiplication at high MOI [20, 21]. The bacteriophages could effectively self-replicate at the optimum MOI of 1.

The specificity of P22 to antibiotic-sensitive *S*. Typhimurium ATCC 19585 was not different from that to ciprofloxacin-induced antibiotic-resistant *S*. Typhimurium ATCC 19585 (Fig. 5). The specificity of P22 to *S*. Typhimurium ATCC 19585 was not changed after the induction of antibiotic resistance. The observation implies that P22 can be used as biocontrol agent against multidrug-resistant *S.* Typhimurium. Bacteriophages have renewed attention to potential alternative over conventional antibiotics [22]. However, the lowest lytic efficacy of bacteriophages against the clinically isolated antibiotic-resistant *S*. Typhimurium CCARM 8009 might be due to the alteration in receptors, leading to the decrease in binding affinity [23]. The specificity of bacteriophages against host cells is highly associated with the binding affinity between the receptors in the host and receptor-binding proteins in bacteriophages [10, 24]. The alterations in host cell surface receptors are responsible for bacteriophage resistance, resulting in the reduced lytic activity [12, 25].

## Conclusions

This study highlights the possibility of using bacteriophages for biocontrol of multidrug-resistant *S.* Typhimurium. The most significant finding of this study was that the ciprofloxacin-induced antibiotic-resistant *S*. Typhimurium ATCC 19585 was effectively inactivated by P22, but the clinically isolated antibiotic-resistant *S*. Typhimurium CCARM 8009 was resistant to P22 due to the alteration in bacteriophage-binding receptors. PBST10, PBST13, PBST32, and PBST35 showed the lytic activity against the clinically isolated antibiotic-resistant *S*. Typhimurium CCARM 8009. The results would open the door for developing new bacteriophage control system against multidrug-resistant pathogens. However, in order to use bacteriophage as a potential alternative agent against multidrug-resistant *S.* Typhimurium, further study is needed to elucidate the alterations in bacteriophage-binding receptors in association with the lytic efficacy of bacteriophages.

## Abbreviations

MOI: multiplicity of infection
TSB: trypticase soy broth
CFU: colony-forming unit
PFU: plaque-forming unit
PEG: polyethylene glycol
TEM: transmission electron microscope
ATCC: American Type Culture Collection
KCCM: Korean Culture Center of Microorganism
AMP: ampicillin
CEF: cefotaxime
CEP: cephalothin
CHL: chloramphenicol
CIP: ciprofloxacin
KAN: kanamycin
LEV: levofloxacin
MER: meropenem
NAL: nalidixic acid
NOR: norfloxacin
SMA/TMP: sulfamethoxazole/trimethoprim
TET: tetracycline
